# New Appliances and Things Medical

**Published:** 1894-02-10

**Authors:** 


### NEW APPLIANCES AND THINGS MEDICAL.

[All preparations, appliances, novelties, &c., of which a notice is desired, should be sent for the Editor, to care of The Manager, 428, Strand, London, W.C.]

### KINGZETT'S IMPROVED SULPHUR FUMIGATING CANDLES.

Kingzett's Sulphur Fumigating Candles have already been described in these columns. Our readers may remember that Mr. Kingzett's first patent consisted in the employment of a fuse moulded in the form of a cone, which was set in or on a mass of sulphur moulded in a suitably shaped vessel. The fuse in question consisted of sulphur in admixture with chlorate or nitrate of potassium or sodium. Further investigation has proved that such a fuse may be altogether dispensed with and in the present candles a strip or ribbon of Brussels net, or other similar material, is employed, which is first of all coated with a thin layer of sulphur by passage through a bath of that substance in a molten state. The strips of material thus coated with pure sulphur are afterwards cut up into suitable lengths, bent into circular form, and then insetted in the molten mass of sulphur forming the body of the candle. On application of a light to the prepared strip, which stands up above the surface of the body of the candle, it immediately takes fire and burns with great rapidity, the molten sulphur running down on to the surface of the candle and firing it immediately. The inventor has found that many materials may be employed, such as muslin and tissue paper, but Brussels net is better than all others, because it gives very little ash, and the air spaces in the fabric, which are revealed immediately that it is ignited, serves to feed the burning sulphur with a plentiful supply of atmospheric oxygen. We understand that Kingzett's Sulphur Candles, in the two forms that we have now described, have been adopted for use by many of the sanitary authorities in this country for the fumigation of infected rooms and dwellings, and they may be also very usefully employed for the extermination of insect pests, besides which they are suitable for use in many particular trades; thus, for example, by brewers for fumigation of their vats and tuns in order to free them from troublesome secondary ferments; while, again, for example, they can be most usefully employed for the purification of slaughter-houses and butchers' store rooms. Whenever sulphur is used for the purposes of fumigation, it should be remembered that the products of its combustion act much more efficiently when the atmosphere is moist; and, in any case, where this condition of moisture is not already present, it is well to previously saturate the air as much as possible with a steam kettle or by other means.

### DR. CHAMPETIER DE RIBE'S BAGS.

Messrs. Allen and Hanbury write to point out that the bag of which we gave an account last week, is a modification of that invented by Dr. Champetier de Ribe, "with improvements as suggested by Dr. W. McD. Ellis."

